# Analysis of the characteristics of spino-pelvic sagittal parameters in Degenerative Thoracolumbar Kyphosis deformity and the mechanism of sagittal imbalance

**DOI:** 10.1371/journal.pone.0354693

**Published:** 2026-07-30

**Authors:** Ruifeng Xun, Liang Zhao, Weixing Han, Gang Chen, Wei Zhang, Kangkang Wang, Xiwen Sun, Qiang Zhang, Yunlei Zhai, Haiyang Yu, Xilong Cui

**Affiliations:** 1 Department of Orthopedics, Fuyang People’s Hospital Affiliated to Anhui Medical University, Fuyang, Anhui, China; 2 Department of Orthopaedics, Linquan County People’s Hospital, Fuyang, Anhui, China; 3 Spinal Deformity Clinical Medicine and Research Center of Anhui Province, Fuyang, Anhui, China; 4 Xiaogan First People’s Hospital, Xiaogan, Hubei, China; University of Perugia: Universita degli Studi di Perugia, ITALY

## Abstract

**Background:**

Degenerative thoracolumbar kyphosis (DTLK) is a subtype of adult spinal deformity. If DTLK progresses further, it can develop into global kyphosis (GK). Although numerous studies have described the sagittal parameters of the spino-pelvic in adult spinal deformities, there is currently no report on the sagittal parameters of DTLK. To date, there have been no reports that elucidate the mechanism of sagittal imbalance by comparing it with sagittal balance, or by examining the sagittal parameters of healthy individuals. To address this gap, the study employed a retrospective analysis, measuring parameters such as the sagittal vertical axis (SVA), thoracic kyphosis (TK), thoracolumbar kyphosis (TLK), lumbar lordosis angle (LL), upper arc of total lumbar lordosis (UALL), lower arc of total lumbar lordosis (LALL), pelvic incidence (PI), pelvic tilt (PT), sacral slope (SS), spino-sacral angle (SSA), and PI-LL. This study aims to clarify the occurrence and compensatory mechanisms of sagittal balance and sagittal imbalance in patients with DTLK.

**Objective:**

To investigate the spino-pelvic sagittal parameters in patients with degenerative thoracolumbar kyphosis and the compensatory mechanisms for the occurrence of sagittal imbalance.

**Method:**

Our research is a cross-sectional study. The imaging data used in this study were extracted on August 15, 2025. A retrospective analysis was conducted on the preoperative imaging data of 339 patients diagnosed with DTLK at Fuyang People’s Hospital from 2016 to 2025. The sagittal parameters of the spino-pelvic were measured using Surgimap software and compared to those of healthy individuals as documented in the literature. The data were divided into a balanced group and an imbalanced group based on SVA and the spino-pelvic parameters of healthy individuals. The balanced group was then compared with the imbalanced group.

**Results:**

The spino-pelvic sagittal parameters of DTLK patients were significantly different from those of healthy individuals. In the balanced group, TK, TLK, LL, and PT were higher than those of normal individuals, while SS, PI, PI-LL, SSA, and UALL were lower than those of normal individuals. Compared with the imbalanced group, the balanced group had higher TK, TLK, LL, LALL, and SSA and lower SS, PT, PI-LL, and UALL. SVA showed significant correlations with multiple sagittal parameters.

**Conclusions:**

DTLK patients exhibit significant differences from healthy individuals in various sagittal parameters. Balance is primarily maintained through the posterior tilt of the pelvis and a reduction in the lumbar lordosis to compensate. When an imbalance occurs, the LL, LALL, UALL, PT, and PI-LL increase as compensatory mechanisms for the loss of balance. Once the UALL is exhausted, compensation shifts to an anterior convexity of the lower lumbar curve and a posterior rotation of the pelvis.

## Introduction

Degenerative thoracolumbar kyphosis (DTLK) is defined as a thoracolumbar kyphosis (TLK) angle of 15° or greater accompanied by degenerative changes of the spine. If the disease progresses further, it can transform into global kyphosis (GK). DTLK is one of the common subtypes of adult spinal deformities that significantly affects the quality of life. Its pathological process involves intervertebral disc degeneration, wedge-shaped deformation of the vertebrae, and loss of the sagittal plane balance [1,2]. Spinal degenerative diseases are commonly observed in middle-aged and elderly women [3–5]. This disease is characterized by features such as overall forward tilting, posterior tilting of the pelvis, and the absence of lumbar lordosis. Unlike degenerative lumbar spondylolisthesis or simple thoracic kyphosis, DTLK is characterized by a pathological process involving multi-segmental intervertebral disc degeneration, vertebral wedge deformation, and spinal-pelvic misalignment, leading to typical clinical manifestations: kyphotic posture, back pain, and impaired standing balance. These symptoms fundamentally differ from the localized presentations of spondylolisthesis or the compensatory mechanisms seen in isolated thoracic kyphosis. In the Roussouly classification, type Ⅱ spinal deformities, which are characterized by a flat back, are most likely to develop into DTLK [6]. Multiple studies have shown that sagittal imbalance can cause patients to experience back pain and difficulty standing, thereby significantly reducing the health-related quality of life [7–11].

Previous literature on spinal-pelvic sagittal parameters has primarily focused on adult spinal deformities, with most research concentrating on deep learning and improvements in measurement techniques [12–15]. Although Liu C et al. analyzed sagittal parameter characteristics in balanced versus imbalanced DTLK patients, their study primarily focused on DTLK subtype classification, and comparative analyses of sagittal parameters specific to this subtype remain limited [16]. More importantly, these studies did not comprehensively compare DTLK patients with healthy control populations, nor did they provide a detailed analysis of parameter differences between balanced and imbalanced subgroups. This knowledge gap limits clinicians’ understanding of the compensatory mechanisms that enable sagittally balanced patients to maintain equilibrium, and also hinders identification of the key parameters involved in the progression of sagittal imbalance. Therefore, this study aims to compare spinal-pelvic sagittal parameters between DTLK patients and healthy individuals, and between sagittally balanced and imbalanced subgroups, in order to elucidate the compensatory mechanisms underlying the maintenance or loss of sagittal balance.

## Methods

### Patient recruitment and inclusion/exclusion criteria

This is a retrospective cross-sectional study. Imaging data of patients with DTLK were retrospectively collected. The cases were selected from the imaging data of 339 patients with degenerative thoracolumbar kyphosis who were treated at the Orthopedics Department of Fuyang People’s Hospital in Anhui Province from July 2016 to July 2025. Among them, there were 95 males and 244 females. The data for the normal and asymptomatic population were obtained from the literature [17,18]. Inclusion criteria: 1) Diagnosed with degenerative thoracolumbar kyphosis (TLK ≥ 15° on standing radiographs; presence of back pain, postural deformity, or impaired standing balance; and degenerative changes on imaging excluding congenital, infectious, traumatic, or inflammatory etiologies) [19]. 2) Eligible patients had full-length standing anteroposterior and lateral spinal radiographs available for analysis. Exclusion criteria: 1) Incomplete imaging data from full-length spinal anteroposterior and lateral radiographs; 2) A previous history of spinal, pelvic, or hip joint surgeries, accompanied by severe hip and knee joint diseases; 3) Kyphosis caused by spinal tuberculosis, congenital spinal deformity, ankylosing spondylitis, etc.; 4) Complicated with other spinal disorders, such as lumbar spondylolisthesis, isthmic spondylolysis, etc.; 5) Presence of spinal fractures, tumors, or infections.

### Ethics approval and consent to participate

This study received approval from the Institutional Review Board and the Ethics Committee of Fuyang People’s Hospital Affiliated to Anhui Medical University (No.2024−223). As this was a retrospective study using existing de-identified imaging data collected during routine clinical care, the ethics committee waived the requirement for written informed consent. This research adheres to the ethical standards for medical research involving human subjects as stipulated in the Declaration of Helsinki.

### Sample size rationale

The sample size was determined by the number of eligible patients who met the inclusion criteria during the study period (July 2016 to July 2025). Specifically, a total of 412 patients with DTLK were initially identified from the institutional imaging database. After applying the exclusion criteria, 73 patients were excluded (43 with incomplete imaging data, 12 with prior spinal surgery, 18 with spinal tuberculosis or tumors), leaving 339 patients for final analysis. No a priori sample size calculation was performed, consistent with the exploratory and descriptive nature of this cross-sectional study. The observed effect size for the primary comparison of sagittal vertical axis(SVA) between the balanced and imbalanced groups was very large (Cohen’s d = 1.82), indicating that the sample size was more than adequate to detect clinically meaningful differences.

### Measurement method of image data

The 339 collected full-length anteroposterior and lateral spinal radiographs were imported into Surgimap software for the measurement of spino-pelvic sagittal parameters [20]. The parameters were measured by two experienced spinal surgeons, and the average value was calculated. Standard spinopelvic sagittal parameters were defined as previously described [21–23].

Sagittal vertical axis (SVA): horizontal distance from the C7 plumb line to the posterior superior corner of the sacral endplate [21,22]. Thoracic kyphosis (TK): Cobb angle from T4 to T12 [23]. Thoracolumbar kyphosis (TLK): Cobb angle from T10 to L2 [21]. Lumbar lordosis (LL): Cobb angle from L1 to S1 [21]. Upper arc of lumbar lordosis (UALL): angle between L1 and L4 [24]. Lower arc of lumbar lordosis (LALL): angle between L4 and S1 [24]. For LL, LALL, and UALL, negative values indicate lordosis, and positive values indicate kyphosis.

Pelvic incidence (PI): angle between the line to the bicoxofemoral midpoint and the perpendicular to the S1 upper endplate [21]. Pelvic tilt (PT): angle between the same line and the vertical plumb line [21]. Sacral slope (SS): angle between the S1 upper endplate and the horizontal line [21]. Spino-sacral angle (SSA): angle formed by the C7‑sacral line and the sacral endplate line [21]. PI‑LL: Pelvic incidence minus lumbar lordosis.

### Reliability assessment

To evaluate measurement reliability, a random subset of 30 patients was selected for inter-observer and intra-observer reliability analyses. For inter-observer reliability, both surgeons independently measured all parameters in this subset without knowledge of each other’s results. For intra-observer reliability, one surgeon repeated the measurements after a 4-week interval, blinded to the initial results. Intraclass correlation coefficients (ICC) with 95% confidence intervals were calculated using a two-way random-effects model for absolute agreement.

### Variables definition

The following variables were defined a priori. Outcome variable: SVA, used to classify patients into the balanced group (SVA ≤ 5 cm) and imbalanced group (SVA > 5 cm) [25]. Exposure variables: TK, TLK, LL, UALL, LALL, PI, PT, SS, SSA, and PI-LL. Potential confounders: age, sex, and body mass index (BMI), which were compared between groups to assess baseline comparability.

### Potential sources of bias

To minimize selection bias, all consecutive patients diagnosed with DTLK at our institution between July 2016 and July 2025 were considered for inclusion. To reduce measurement bias, all radiographic parameters were measured independently by two spine surgeons who were blinded to the patients’ group classification. The average of the two measurements was used for analysis. To mitigate information bias, patients with incomplete full-length spinal radiographs or missing key clinical data were excluded a priori according to the pre-specified exclusion criteria. Confounding bias was addressed by comparing baseline characteristics (age, sex, BMI) between the balanced and imbalanced groups; no significant differences were observed, indicating adequate comparability.

### Statistical analysis

This study retrospectively analyzed the correlations among spino-pelvic sagittal parameters in DTLK patients. Based on SVA values, all imaging data were categorized into the balanced group (SVA ≤ 5 cm) and the imbalanced group (SVA > 5 cm). Data statistical analysis was conducted using SPSS version 25.0. Descriptive analysis was performed for age and various parameters. Normality tests were conducted on all data, and those that followed a normal distribution were expressed as mean ± standard deviation. For comparisons between DTLK patients and published normative data from healthy individuals, one-sample t-tests were employed. For comparisons between the balanced and imbalanced groups, independent samples t-tests were used for continuous variables, and chi-square tests were used for categorical variables (sex). The correlation between each parameter was analyzed using the Pearson correlation test. The intra-class correlation coefficient (ICC) with 95% confidence interval was run to assess intra- and inter-rater agreement of radiographic parameters of interest. An ICC value of higher than 0.90 is defined as excellent agreement between the two measurements. A P-value less than 0.05 indicated statistically significant differences.

## Result

A total of 339 patients with DTLK were included in this study, including 95 males and 244 females. Baseline characteristics are presented in Table 1. There were no significant differences in age (p = 0.247), sex (p = 0.642) distribution, and BMI (p = 0.431) between the balanced group and the imbalanced group. The ICCs of radiographic parameter values were higher than 0.9 for intra- and inter-observer agreement (Table 2).

**Table 1:** Baseline characteristics of all participants, balanced group, and imbalanced group.

Variable	Total (n = 339)	Balanced group (n = 189)	Imbalanced group (n = 150)	P-value
Age (years)	67.95 ± 7.22	67.36 ± 6.88	68.65 ± 7.61	0.247
Sex (Male/Female)	95/244	51/138	44/106	0.642
BMI (kg/m²)	24.29 ± 3.12	23.96 ± 3.27	24.56 ± 3.58	0.431

**Table 2:** Inter-observer and intra-observer reliability of spino-pelvic sagittal parameter measurements.

Radiographic Measure	Intra-observer ICC (95% CI)	Inter-observer ICC (95% CI)
SVA(cm)	0.94 (0.74–0.98)	0.98 (0.88–0.99)
TK(°)	0.99 (0.98–0.99)	0.99 (0.97–0.99)
TLK(°)	0.98 (0.97–0.99)	0.96 (0.92–0.98)
LL(°)	0.99 (0.99–0.99)	0.97 (0.95–0.98)
LALL(°)	0.95 (0.90–0.97)	0.95 (0.91–0.97)
UALL(°)	0.96 (0.92–0.98)	0.93 (0.85–0.96)
PT(°)	0.94 (0.88–0.97)	0.93 (0.87–0.97)
PI(°)	0.97 (0.95–0.98)	0.97 (0.87–0.97)
SS(°)	0.95 (0.90–0.97)	0.94 (0.88–0.97)
PI-LL(°)	0.98 (0.96–0.99)	0.95 (0.89–0.97)
SSA(°)	0.94 (0.89–0.97)	0.91 (0.82–0.95)

The comparison of sagittal parameters of the spino-pelvic between 339 patients with degenerative thoracolumbar kyphosis and healthy middle-aged and elderly individuals revealed that the SVA, TK, TLK, LL, LALL, PT, PI-LL, and UALL of the patients with degenerative spinal kyphosis were significantly higher than those of the normal population, whereas SS, PI, and SSA were significantly lower (all P < 0.001) (Table 3).

**Table 3:** Comparison of spino-pelvic parameters between DTLK patients and normal population.

Parameters			SVA	TK	TLK	LL	LALL	PT	PI	SS	PI-LL	SSA	UALL
This study		Average	49.99	38.38	31.8	−30.1	−34.95	23.38	44.44	21.05	13.38	106.64	4.77
		SD	61.44	18.60	13.04	23.00	13.09	12.50	12.05	12.77	21.62	16.77	16.87
Kang KB,et al		Average	−0.4	30.1	9.5	−57.4	−39.1	11.2	48.2	37	9.2	/	−18.3
		SD	2.7	8.7	7.5	8.5	6.6	/	7.9	6.4	/	/	/
Zeng Z,et al		Average	/	/	/	/	/	/	/	/	/	122.9	/
		SD	/	/	/	/	/	/	/	/	/	7.8	/
Statistical results	t	/	15.09	8.30	31.02	21.85	5.79	−5.84	17.94	−23.02	/	3.363	/
	P	/	<0.001	<0.001	<0.001	<0.001	<0.001	<0.001	<0.001	<0.001	<0.001	<0.001	<0.001

**Fig 1:**
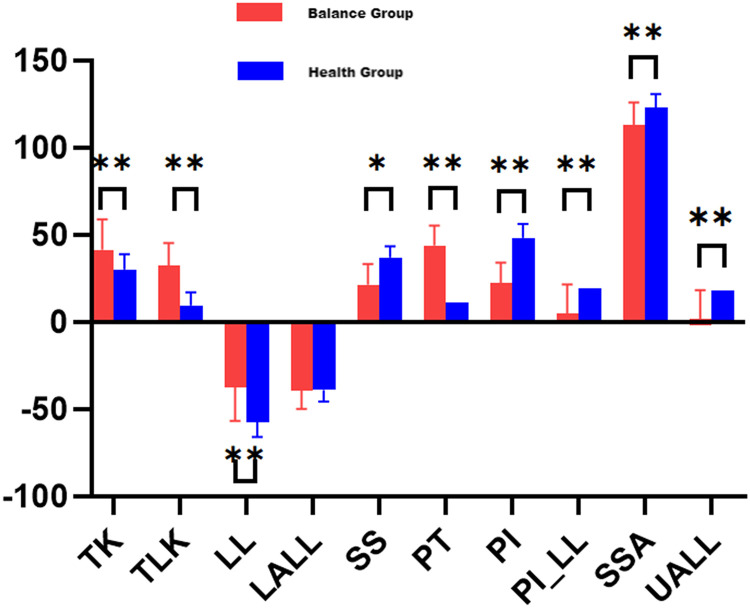
Comparison of spino-pelvic sagittal parameters between the balanced group and normal population. The balanced group exhibited significantly higher TK, TLK, LL, and PT, and significantly lower SS, PI, PI-LL, SSA, and UALL compared to the normal population (*p < 0.05; **p < 0.001).

The patients were divided into two groups: the balanced group and the imbalanced group. When compared with the normal middle-aged and elderly population regarding sagittal parameters, as illustrated in Fig 1, the balanced group exhibited higher values for TK, TLK, LL, and PT than the normal population (all P < 0.001). Conversely, the balanced group had significantly lower values for SS (p < 0.05), PI (p < 0.001), PI-LL (p < 0.001), and the UALL (p < 0.001) compared to the normal population. Compared to the imbalanced group, the TK and SSA in the balanced group were significantly higher (all p < 0.001), while LL (p < 0.001), LALL (p < 0.001), UALL (p < 0.001), SS (p = 0.03), PT (p < 0.002), and PI-LL (p < 0.001) were significantly lower. There was no statistical significance in the gender (p = 0.642), age (p = 0.247), and BMI (p = 0.431) between the two groups (Tables 1 and 4).

**Table 4:** Comparison of sagittal spino-pelvic parameters between the balanced group and the imbalanced group.

Parameters			TK^**^	TLK	LL^**^	LALL^**^	SS^*^	PT^**^	PI	PI_LL^**^	SSA^**^	UALL^**^
Balanced		Average	41.27	32.25	−37.64	−39.33	21.44	43.88	22.44	4.93	112.94	1.68
		SD	17.52	13.14	19.13	10.51	11.78	11.52	11.60	16.70	13.09	16.62
Imbalance		Average	34.84	31.20	−20.76	−29.42	25.70	45.11	19.41	23.81	98.71	8.67
		SD	19.38	12.96	24.04	13.98	12.94	12.72	13.94	22.48	17.55	16.43
Statistical results	t	/	10.42	0.66	51.90	56.05	4.70	10.03	0.90	79.13	73.07	19.92
	p	/	0.001	0.42	<0.001	<0.001	0.03	<0.002	0.34	<0.001	<0.001	<0.001

* indicates a statistically significant difference between the imbalanced group and the balanced group，*indicating p < 0.05 and **indicating P < 0.001.

The correlation analysis of the spino-pelvic sagittal parameters revealed that the SVA correlated with TK (r = −0.19, P < 0.001), LL (r = 0.50, P < 0.001), LALL (r = 0.46, P < 0.001), PT (r = 0.29, P < 0.001), PI (r = 0.16, P < 0.001), SS (r = −0.13, P = 0.01), PI-LL (r = 0.62, P < 0.001), SSA (r = −0.59, P < 0.001), and UALL (r = 0.32, P < 0.001). TK correlated with TLK (r = 0.32, P < 0.001), LL (r = −0.62, P < 0.001), LALL (r = −0.47, P < 0.001), PI (r = 0.20, P < 0.001), SS (r = 0.29, P < 0.001), PI-LL (r = −0.56, P < 0.001), SSA (r = 0.30, P < 0.001), and UALL (r = −0.48, P < 0.001). TLK correlated with LALL (r = −0.39, P < 0.001), PT (r = 0.11, P = 0.040), SS (r = −0.21, P < 0.001), SSA (r = −0.18, P < 0.001), and UALL (r = 0.30, P < 0.001) (Table 5).

**Table 5:** Correlation of spino-pelvic parameters in degenerative thoracolumbar kyphosis based on pearson test.

Parameters	Statistical	TK	TLK	LL	LALL	PT	PI	SS	PI_LL	SSA	UALL
SVA	R	−0.19	0.03	0.50	0.46	0.29	0.16	−0.13	0.62	−0.59	0.32
	P	0.00	0.53	0.00	0.00	0.00	0.00	0.01	0.00	0.00	0.00
TK	R	1.00	0.32	−0.62	−0.47	−0.10	0.20	0.29	−0.56	0.30	−0.48
	P		0.00	0.00	0.00	0.07	0.00	0.00	0.00	0.00	0.00
TLK	R		1.00	0.00	−0.39	0.11	−0.10	−0.21	−0.09	−0.18	0.30
	P			0.98	0.00	0.04	0.05	0.00	0.09	0.00	0.00
LL	R			1.00	0.69	0.48	−0.34	−0.79	0.83	−0.86	0.83
	P				0.00	0.00	0.00	0.00	0.00	0.00	0.00
LALL	R				1.00	0.37	−0.21	−0.56	0.63	−0.67	0.16
	P					0.00	0.00	0.00	0.00	0.00	0.00
PT	R					1.00	0.46	−0.55	0.76	−0.56	0.36
	P						0.00	0.00	0.00	0.00	0.00
PI	R						1.00	0.49	0.17	0.30	−0.30
	P							0.00	0.00	0.00	0.00
SS	R							1.00	−0.58	0.83	−0.64
	P								0.00	0.00	0.00
PI-LL	R								1.00	−0.75	0.63
	P									0.00	0.00
SSA	R									1.00	−0.65
	P										0.00

## Discussion

This study identified prominent disparities in spinopelvic sagittal alignment between patients with DTLK and healthy volunteers. Within the sagittal balanced subgroup, TK, TLK, LL, and PT were consistently greater than corresponding values in asymptomatic individuals. Relative to the sagittal imbalanced subgroup, the balanced cohort also displayed higher measurements of TK, TLK, LL, LALL, and SSA.

Our results indicated that the SVA, TK, TLK, LL, LALL, PT, PI-LL, and UALL values of DTLK patients were significantly higher than those of the normal controls, whereas SS and SSA were significantly lower. Yukawa Y, et al. [26]evaluated the lumbar sagittal parameters in a large number of asymptomatic individuals using X-ray films. They conducted a detailed analysis of the sagittal parameters in the asymptomatic population and found that the core characteristics of degenerative kyphosis are global anterior tilt, pelvic posterior tilt, and the absence of lumbar lordosis. The overall sagittal parameters of the DTLK patients reported in our study align with the conclusions of numerous previous studies conducted both domestically and internationally [26–28]. However, our sample size is much larger. This outcome further corroborates the characteristic changes of DTLK in the sagittal plane balance.

To investigate the spinal-pelvic compensatory mechanism enabling some DTLK patients to maintain balance in the sagittal plane, we found that among patients with DTLK balance in the sagittal plane, sagittal parameters such as TK, TLK, LL, and PT were all higher than those in healthy individuals, whereas SS and UALL were lower. This indicates that the reduction of UALL serves as an active extension compensation for DTLK, and the increase in the anterior curvature angle of the upper lumbar curve can counteract the tendency of the posterior convexity to cause an anterior sagittal inclination of the spine. Studies have shown that a reduction in the anterior curvature of the upper lumbar spine is negatively correlated with the SVA, suggesting that the anterior curvature angle of the upper lumbar spine plays a compensatory role in maintaining sagittal position balance [29,30]. An increase in PT signifies that the trunk rotates through the pelvis to shift the center of gravity backward, thereby further reducing the load on the anterior curvature of the spine. The increase in the pelvic inclination angle is equivalent to a posterior tilt of the pelvis, causing the body’s center of gravity to shift backward and reducing the forward shear force generated by the anterior tilt of the spine. This represents a classic compensatory mechanism involving pelvic rotation [31–33]. In this study, TK exhibited an increasing trend of kyphosis. This rise in the thoracic kyphosis angle may be associated with the local stiffness resulting from degenerative thoracolumbar kyphosis or the absence of compensatory thoracic curvature that has not yet developed. The lack of flexibility restricts the extension compensation of the thoracic spine during the early stages of degenerative kyphosis [34]. Studies have reported that when the thoracic spine can fully extend, patients typically only require a mild posterior pelvic tilt to maintain balance. If thoracic spine extension is limited, the compensation shifts to the lower limbs and pelvis, resulting in insufficient hip extension and increased knee flexion, thereby increasing the load on the lower limbs [35–37]. At this point, the anterior convexity compensation of the UALL results in a further increase in the posterior convexity angle of the thoracic segment.

By comparing the sagittal spino-pelvic parameters between the balanced and imbalanced groups in DTLK, we found that the TK level was lower in the imbalanced group compared to the balanced group. Additionally, the levels of LL, LALL, UALL, SS, PT, PI-LL, and SSA were significantly elevated in the imbalanced group. This indicates that following the severe sagittal imbalance of the thoracolumbar lordosis, the body will successively experience exhaustion of the active extension in the anterior curvature of the upper lumbar spine, exhaustion of the extension of the lumbar lordosis, and exhaustion of the compensatory rotation of the pelvis. At this point, the sole compensatory method for maintaining trunk balance is through the thoracic vertebrae. However, the efficacy of this compensation relies on the spinal flexibility of the DTLK patients. These findings are similar to those of Liu et al. regarding the sagittal compensatory pattern of ankylosing spondylitis. Specifically, before compensation is exhausted, the body tends to maintain balance by extending the upper lumbar segment (increasing thoracic kyphosis). After compensation is exhausted, it relies on the lordosis of the lumbar segment and the posterior tilt of the pelvis [38]. Studies have shown that in the assessment of changes in thoracic curvature in patients following lumbar lordosis correction surgery, pelvic retroversion and a reduction in thoracic kyphosis are the primary compensatory patterns when lumbar lordosis is inadequate [39]. This, to some extent, explains why, when the anterior-posterior curvature of the spine reaches its limit and cannot be compensated, the spine maintains its sagittal position balance by relying on the posterior tilt of the pelvis and the reduction of the posterior curvature of the thoracic vertebrae.

Upon further summarizing the differences in compensatory mechanisms among healthy individuals, the balanced group, and the imbalanced group, we infer that the overall sagittal compensatory pattern likely represents a progression from primary compensation followed by secondary compensation. The primary compensation refers to the extension of the anterior convexity of the upper lumbar spine. In patients with relatively preserved sagittal balance, the main compensatory mechanism is the reduction of the UALL, that is, the increase of the anterior convex angle of the UALL. The characteristics of the sagittal parameters in this group are relatively low LL, LALL, and UALL. In patients with sagittal imbalance, as observed in the imbalanced group, LL, LALL, and UALL are increased. With upper lumbar curvature exhaustion, the spinal sagittal compensation appears to shift to a mode primarily dominated by pelvic posterior tilt compensation. At this stage, the primary sagittal parameters of the spino-pelvic show the following changes: PT and PI-LL significantly increased, while SS significantly decreased. These variations indicate that during clinical assessment and surgical planning, an individualized analysis must be conducted, taking into account the specific location of kyphosis and spinal flexibility. We believe that progressive loss of lumbar lordosis (LL, LALL shifting toward neutral or kyphotic values), or rapid increases in PT, can be considered early indicators of compensatory exhaustion. This suggests the necessity for early intervention to prevent long-term functional impairment and pain resulting from progressive pelvic posterior tilt.

This study has several limitations. First, the cross-sectional nature prevents the establishment of causality; the compensatory sequence is inferred from group comparisons rather than longitudinal observation. Second, the retrospective, single-center design introduces selection bias and limits generalizability. Third, using historical controls from the literature may introduce spectrum bias and population differences due to differing ethnic backgrounds, age distributions, radiographic protocols, and measurement standards between the present study cohort and the healthy control population, which may reduce the comparability of results. Fourth, missing data bias cannot be excluded because patients with incomplete imaging data were excluded. Finally, static radiographs alone may underestimate the true compensatory range, and unmeasured factors (lower limb compensation, muscle quality) may confound the results.

## Conclusion

The sagittal parameters of the DTLK patients exhibited significant abnormal characteristics. In different equilibrium states, they displayed a stepwise compensatory process, beginning with the anterior convexity of the upper lumbar curve, extending to the lumbar lordosis, and then to the posterior tilt of the pelvis. This compensatory pathway is closely related to the flexibility of the thoracolumbar lordosis, the patient’s age, and the shape of the pelvis. Clinically, especially when focusing on PI-LL matching and PT normalization, it can significantly improve the quality of life of patients while ensuring surgical safety. In the future, through multi-center, dynamic imaging, and rehabilitation intervention methods, the assessment and intervention strategies of the compensatory mechanism should be further refined.

## Abbreviations

DTLK: degenerative thoracolumbar kyphosis
GK: Global kyphosis
SVA: Sagittal vertical axis
TK: Thoracic kyphosis
TLK: Thoracolumbar kyphosis
LL: Lumbar lordosis angle
UALL: Upper arc of total lumbar lordosis
LALL: Lower arc of total lumbar lordosis
PI: Pelvic incidence
PT: Pelvic tilt
SS: Sacral slope
SSA: Spino-sacral angle
